# A Novel Small RNA on the *Pseudomonas putida* KT2440 Chromosome Is Involved in the Fitness Cost Imposed by IncP-1 Plasmid RP4

**DOI:** 10.3389/fmicb.2020.01328

**Published:** 2020-06-23

**Authors:** Hibiki Kawano, Chiho Suzuki-Minakuchi, Daisuke Sugiyama, Natsuki Watanabe, Yurika Takahashi, Kazunori Okada, Hideaki Nojiri

**Affiliations:** ^1^Biotechnology Research Center, The University of Tokyo, Tokyo, Japan; ^2^Collaborative Research Institute for Innovative Microbiology, The University of Tokyo, Tokyo, Japan; ^3^Biotechnology Research Center and Department of Biotechnology, Toyama Prefectural University, Toyama, Japan

**Keywords:** compensatory mutation, experimental evolution, fitness cost, horizontal gene transfer, plasmid, *Pseudomonas*, stress response

## Abstract

Plasmids can provide advantageous traits to host bacteria, although they may impose a fitness cost. Chromosome-encoded factors are important for regulating the expression of genes on plasmids, and host chromosomes may differ in terms of their interactions with a given plasmid. Accordingly, differences in fitness cost loading and compensatory co-evolution may occur for various host chromosome/plasmid combinations. However, the mechanisms of compensatory evolution are highly divergent and require further insights. Here, we reveal novel evolutionally mechanisms of *Pseudomonas putida* KT2440 to improve the fitness cost imposed by the incompatibility P-1 (IncP-1) multidrug resistance plasmid RP4. A mixed culture of RP4-harboring and -free KT2440 cells was serially transferred every 24 h under non-selective conditions. Initially, the proportion of RP4-harboring cells decreased rapidly, but it immediately recovered, suggesting that the fitness of RP4-harboring strains improved during cultivation. Larger-sized colonies appeared during 144-h mixed culture, and evolved strains isolated from larger-sized colonies showed higher growth rates and fitness than those of the ancestral strain. Whole-genome sequencing revealed that evolved strains had one of two mutations in the same intergenic region of the chromosome. Based on the research of another group, this region is predicted to contain a stress-inducible small RNA (sRNA). Identification of the transcriptional start site in this sRNA indicated that one mutation occurred within the sRNA region, whereas the other was in its promoter region. Quantitative reverse-transcription PCR showed that the expression of this sRNA was strongly induced by RP4 carriage in the ancestral strain but repressed in the evolved strains. When the sRNA region was overexpressed in the RP4-free strain, the fitness decreased, and the colony size became smaller. Using transcriptome analysis, we also showed that the genes involved in amino acid metabolism and stress responses were differentially transcribed by overexpression of the sRNA region. These results indicate that the RP4-inducible chromosomal sRNA was responsible for the fitness cost of RP4 on KT2440 cells, where this sRNA is of key importance in host evolution toward rapid amelioration of the cost.

## Introduction

Plasmids are mobile genetic elements that can confer advantages, such as antibiotic resistance and xenobiotic degradation capability, to their hosts via horizontal gene transfer (Top and Springael, 2003; Wiedenbeck and Cohan, 2011). Understanding the mechanisms underlying the behavior of the plasmids themselves, and the strains that harbor them, is essential because plasmid carriage can promote the rapid adaptation of hosts to various environmental conditions (Frost et al., 2005; Gogarten and Townsend, 2005; Anderson and Hughes, 2010). One important factor in determining the behavior of plasmid-harboring strains is the fitness cost imposed by plasmid carriage (Baltrus, 2013; San Millan and MacLean, 2017). Fitness costs can reduce plasmid stability in the host cell (Melling et al., 1977; De Gelder et al., 2007), as well as the survival rate of plasmid-harboring strains in the bacterial consortium (Dale and Smith, 1979).

Generally, chromosome-encoded factors are involved in regulating the transcription of plasmid genes, and the interactions vary among host chromosome/plasmid combinations. The fitness cost is also wholly dependent on these interactions. The plasmid pSf-R27-encoded H-NS-like protein Sfh was reported to be involved in the reduced plasmid fitness cost seen for a *Salmonella enterica* serovar Typhimurium strain (Doyle et al., 2007). Sfh has a stealth function, repressing the expression of plasmid-encoded genes; this is considered important in the plasmid fitness cost. In *Pseudomonas aeruginosa* PAO1, the fitness cost differed markedly according to which of six plasmids were introduced into the strain (San Millan et al., 2018). The metabolic response of PAO1 cells to different plasmids was also characterized, but the mechanism underlying fitness costs remains unclear. The incompatibility P-7 (IncP-7) group plasmid pCAR1 was isolated from *Pseudomonas resinovorans* CA10 (Ouchiyama et al., 1993; Nojiri et al., 2001; Maeda et al., 2003). Previously, we reported that pCAR1 carriage imposed fitness costs differed among three *Pseudomonas* hosts (*P. aeruginosa* PAO1, *Pseudomonas fluorescens* Pf0-1, and *Pseudomonas putida* KT2440) (Takahashi et al., 2015). Moreover, it was demonstrated that pCAR1 carriage affects the chromosomal transcriptional profile differently among hosts (Shintani et al., 2010), and modulates the primary cell functions of each host (Takahashi et al., 2015). Recently, we reported that pCAR1 carriage altered the proteome profile of strain KT2440; in particular, the acylation status of proteins involved in metabolism and translation was altered (Vasileva et al., 2018). These effects of pCAR1 carriage on the host cell may explain the fitness cost imposed by pCAR1.

Previous studies have shown that plasmid-harboring strains can reduce plasmid fitness costs via adaptive evolution (Bouma and Lenski, 1988; Dahlberg and Chao, 2003; Dionisio et al., 2005). Recently, many studies have identified compensatory mutations related to adaptive evolution of chromosomes and/or plasmids. For example, IncP-1 plasmid pBP136 imposed a fitness cost on *Shewanella oneidensis* MR-1, resulting in low stability of pBP136 in MR-1 host cells. However, the stability could be improved by compensatory mutations located either in the replication protein gene on the plasmid or in a transcriptional regulator gene, which was mainly involved in iron metabolism, on the chromosome (Stalder et al., 2017). Also, IncP-1 plasmid RP4 was highly unstable in *Pseudomonas* sp. H2 (Heuer et al., 2007) although compensatory mutations in both helicase-encoding genes and the RNA polymerase β subunit-encoding gene *rpoB* on the chromosome clearly improved plasmid stability (Loftie-Eaton et al., 2017). The non-transmissible plasmid pNUK73 imposed a fitness cost on *P. aeruginosa* PAO1, but compensatory mutations on host helicase or kinase-encoding genes reduced the cost (San Millan et al., 2014). It was also demonstrated that mutations in the *gacA*/*S* two-component regulatory system reduced the fitness cost imposed by plasmid pQBR103 on *P. fluorescens* SBW25 (Harrison et al., 2015). Previously reported mutations involved in the reduction of fitness costs imposed on host cells were found either in protein-encoding regions or their promoter regions, and in transcriptional regulator-encoding genes. Notably, most previous studies that aimed to clarify adaptive evolution employed a single (pure) culture of the plasmid-harboring strain, where the plasmid was somewhat unstable in host cells (Sota et al., 2010; San Millan et al., 2014; Loftie-Eaton et al., 2015, 2017; Stalder et al., 2017). In the experiments, plasmid-free strains occasionally appeared, and the proportion of the plasmid-harboring strain was determined. Therefore, such experiments encompass two events: occasional plasmid loss from the host and survival of the plasmid-harboring strain under competitive culture conditions with plasmid-free strains.

In this study, we examined whether plasmid RP4 imposed a fitness cost on *P. putida* KT2440 (Nelson et al., 2002) by assessing the survival rate in a competitive culture with a plasmid-free KT2440 strain. RP4 is a multidrug resistance plasmid with a broad host range that was isolated from a *P. aeruginosa* strain (Pansegrau et al., 1994). Although RP4 was stably maintained in KT2440 cells under the conditions we employed, RP4-harboring strains were outcompeted by RP4-free strains in the mixed culture condition, indicating that RP4 carriage imposed a severe fitness cost on KT2440 cells. However, the KT2440 strain evolved such that the fitness cost was ameliorated after 144 h of co-cultivation; furthermore, evolved strains outcompeted plasmid-free strains. Through various analyses of the evolved strains, we identified a novel chromosomally encoded small RNA (sRNA) involved in the severe fitness cost imposed by RP4 on KT2440 cells.

## Materials and Methods

### Bacterial Strains, Plasmids, and Growth Conditions

The bacterial strains and plasmids used in this study are listed in Table 1. *P. putida* KT2440 strains were cultivated at 30°C in L broth (LB) (Sambrook and Russell, 2001) or NMM-4 buffer supplemented with 0.1% (w/v) succinate (hereinafter SUC medium) (Shintani et al., 2005a). *Escherichia coli* strains were cultivated at 37°C in LB. For plate cultures, 1.6% (w/v) agar was added to the medium. Ampicillin (Ap; 100 μg/ml), chloramphenicol (Cm; 30 μg/ml), gentamicin (Gm; 120 μg/ml for *Pseudomonas* strains and 15 μg/ml for *E. coli* strains), kanamycin (Km; 50 μg/ml), and/or rifampin (Rif; 25 μg/ml) were added to the selective media.

**TABLE 1:** Bacterial strains and plasmids.

Strains or plasmids	Abbreviation	Relevant characteristics^a^	Source
**Bacterial strains**			
*Escherichia coli* DH5α	–	deoR, *sup*E44, *hsd*R17(rk-, mk+), *pho*A, *rec*A1, *end*A1, *gyr*A96, *thi*-1, *rel*A1, D(*lac*ZYA-*arg*F)U169, f80d*lac*ZDM15, F^–^, l^–^	Toyobo
*E. coli* HB101(RP4)	–	*E. coli* HB101 harboring RP4	This study
*Pseudomonas putida* KT2440	–	Ap^r^, Cm^r^, Sm^r^, Tc^r^	Nelson et al. (2002)
*P. putida* KT2440(RP4)	–	*P. putida* KT2440 harboring RP4	This study
*P. putida* KT2440RG	–	Rif^r^, Gm^r^	Shintani et al. (2005a)
*P. putida* 1-L1	1-L1	Derivatives of *P. putida* KT2440(RP4) randomly isolated from culture after 144-h of competition assay	This study
*P. putida* 1-L2	1-L2	Derivatives of *P. putida* KT2440(RP4) randomly isolated from culture after 144-h of competition assay	This study
*P. putida* 1-L3	1-L3	Derivatives of *P. putida* KT2440(RP4) randomly isolated from culture after 144-h of competition assay	This study
*P. putida* 2-L1	2-L1	Derivatives of *P. putida* KT2440(RP4) randomly isolated from culture after 144-h of competition assay	This study
*P. putida* 2-L2	2-L2	Derivatives of *P. putida* KT2440(RP4) randomly isolated from culture after 144-h of competition assay	This study
*P. putida* 2-L3	2-L3	Derivatives of *P. putida* KT2440(RP4) randomly isolated from culture after 144-h of competition assay	This study
*P. putida* KT2440(pBBR1MCS-5)	KT(vc)	*P. putida* KT2440 harboring pBBR1MCS-5	This study
*P. putida* KT2440(RP4)(pBBR1MCS-5)	KTRP4(vc)	*P. putida* KT2440 harboring RP4 and pBBR1MCS-5	This study
*P. putida* KT2440(pBBR100)	KT(100)	*P. putida* KT2440 harboring pBBR100	This study
*P. putida* KT2440(pBBR200)	KT(200)	*P. putida* KT2440 harboring pBBR200	This study
*P. putida* KT2440(pBBR300)	KT(300)	*P. putida* KT2440 harboring pBBR300	This study
*P. putida* KT2440(pBBR400)	KT(400)	*P. putida* KT2440 harboring pBBR400	This study
**Plasmids**			
RP4	–	Carrying Ap^*r*^, Km^*r*^, and Tc^*r*^ genes, IncP-1 group	Pansegrau et al. (1994)
pT7Blue T-vector	–	Ap^*r*^, *lacZ*	Novagen
pBBR1MCS-5	–	Gm^*r*^, P*_*lacZ*__α_* promoter	Kovach et al. (1995)
pBBR-ox	–	pBBR1MCS-5, containing 6,157,772–6,159,017 region of *P. putida* KT2440	This study
pBBR100	–	pBBR1MCS-5, containing 6,158,582–6,158,681 region of *P. putida* KT2440	This study
pBBR200	–	pBBR1MCS-5, containing 6,158,482–6,158,681 region of *P. putida* KT2440	This study
pBBR300	–	pBBR1MCS-5, containing 6,158,382–6,158,681 region of *P. putida* KT2440	This study
pBBR400	–	pBBR1MCS-5, containing 6,158,282–6,158,681 region of *P. putida* KT2440	This study
pTR8599	–	pT7Blue T-vector, containing 5′-RACE-PCR product amplified by UPM and 8599-RACE-GSP	This study

*^*a*^Ap^*r*^, Cm^*r*^, Gm^*r*^, Sm^*r*^, Tc^*r*^, Rif^*r*^, Km^*r*^ indicate resistance to ampicillin, chloramphenicol, gentamicin, streptomycin, tetracycline, rifampicin, and kanamycin, respectively.*

### Standard DNA Manipulation

Standard methods were used for the extraction of plasmid DNA, DNA digestion with restriction endonucleases, DNA ligation, and transformation of competent *E. coli* cells (Sambrook and Russell, 2001). The primers used in this study are listed in Table 2. Total DNA was extracted from *Pseudomonas* strains using hexadecyltrimethylammonium bromide (Shintani et al., 2005b). Electroporation of *Pseudomonas* was performed according to the method described by Itoh et al. (1994).

**TABLE 2:** Oligonucleotide primers used in this study.

Primer name	Sequence (5′ to 3′)^a^	References
**For colony hybridization**		
RP4_trfA2-F	GAAACACACGAAGCAGCAGA	This study
RP4_trfA2-R	AAACAGCACGACGATTTCCT	This study
**For qRT-PCR**		
univ16S-F	ACACGGTCCAGACTCCTACG	Miyakoshi et al. (2007)
univ16S-R	TACTGCCCTTCCTCCCAACT	Miyakoshi et al. (2007)
PP_5401.2-F	TGGTTCCTTTGTGTGAAGCCTG	This study
PP_5401.2-R	TGAGAAACGTGCCGGTAATGAAG	This study
**For preparation of overexpression vector**		
7772-F-SpeI	TTTACTAGTAGCAGAACCCTCTCAAGCTGTG	This study
9017-R-SalI	TTTGTCGACCGACGGTACCGATACGAATC	This study
8582-F-EcoRI	CCCGAATTCTTTCAGTACGCCAGGATGTC	This study
8482-F-EcoRI	CCCGAATTCGCATAAGCTTCCAGCTCATT	This study
8382-F-EcoRI	TCTGAATTCTATGGGTCGAGGCGATCAAG	This study
8282-F-EcoRI	TCTGAATTCGCTTTCGGGTTCTCAAGGTG	This study
8681-R-SalI	TTTGTCGACGAACGTAGCAACCACCTGTC	This study
**For 5**′**-RACE analysis**		
8599-RACE-GSP	GTCTATGATCCCCTTCATTACCGGCACG	This study
R-20mer	AGCTATGACCATGATTACGC	This study
**For competition assay detected by PCR**		
PP3700FNdeI	CATATGTCCCCATTAGCCGCTGC	Miyakoshi et al. (2007)
PP3700RXbaI	TCTAGACTATAGACGCAGATCAAC	Miyakoshi et al. (2007)
pBBR1MCS-ins-F	ACTCATCGCAGTCGGCCTAT	This study
pBBR1MCS-ins-R	ATGCTTCCGGCTCGTATGTT	This study

*^*a*^Underlined nucleotides represent artificial restriction sites.*

### Preparation of RP4-Harboring KT2440 Strain

The RP4-harboring KT2440 strain was constructed by filter mating between KT2440 and *E. coli* HB101(RP4) on LB agar plates. Filter mating was performed as described previously (Shintani et al., 2005b). Resultant transconjugant candidates were selected on LB agar plates supplemented with both Cm (30 μg/ml) and Km (50 μg/ml). To verify that the isolated colonies were accurate transconjugants, total DNA extracted from the candidates was subjected to polymerase chain reaction (PCR) using the primer sets listed in Table 2. The primer sets were designed to be specific for *trfA2*, encoding a replication initiation protein on plasmid RP4 (RP4_trfA2-F, R), and for *parI* on the KT2440 chromosome (PP3700FNdeI, PP3700RXbaI).

### Competition Assay

For the pair of RP4-harboring and RP4-free KT2440 cells, a competition assay was performed as described previously (Takahashi et al., 2015). Each RP4-harboring and RP4-free strain was cultivated in 100 ml of SUC medium under non-selective conditions on a rotating shaker (120 rpm), and the initial optical density at 600 nm (OD_600_) was adjusted to 0.03. After a 24-h inoculation, the OD_600_ of each culture was equalized with carbon-free (CF) buffer (NMM-4 buffer with no minerals). RP4-harboring and -free cells were mixed equally and diluted 100-fold in SUC medium. After a 24-h mixed cultivation, 1 ml of culture was transferred into 100 ml of fresh SUC medium and incubated again for 24 h. Several transfers were performed. At the time of each transfer, small portions of the culture were spread on LB agar plates after appropriate dilution. After counting the colonies on the LB plates, the proportion of RP4-harboring cells was determined by colony hybridization using probes designed for a 520-bp region within the *trfA2* gene on RP4. Colony hybridization was performed as described previously (Takahashi et al., 2015). The fitness of the plasmid-harboring strain relative to the plasmid-free strain was assessed using W values (Lenski et al., 1991). *W* = ln(*N*_*eachpoint, harboring*_/*N*_*initial, harboring*_)/ln(*N*_*eachpoint, free*_ /*N*_*initial, free*_), where *W* is the fitness of the plasmid-harboring strain, *N*_*eachpoint, harboring*_ and *N*_*initial, harboring*_ are the colony-forming units (CFUs) of plasmid-harboring strains at each point and the initial point of the assay, respectively, and *N*_*eachpoint, free*_ and *N*_*initial, free*_ are the CFUs of plasmid-free strains at each point and the initial point of the assay, respectively.

To determine the fitness of sRNA region-overexpressed strains, equal amounts of overexpressed and vector control strains were mixed, and competitive cultivation was conducted in SUC medium supplemented with Gm. Total DNA was extracted from 20-ml cultures at 0, 48, 96, 144 h after inoculation. First, the *parI* gene (∼1.1 kb) on the KT2440 chromosome from each DNA sample was PCR-amplified. Next, the sRNA region inserted into pBBR1MCS-5 was amplified by PCR. The band size was 500 bp for pBBR1MCS-5 (control), 600 bp for pBBR100, 700 bp for pBBR200, 800 bp for pBBR300, and 900 bp for pBBR400. Band intensity was quantified manually using ImageJ software (NIH, Bethesda, MD, United States). The ratio of the overexpressed strain at each point was calculated as follows: band intensity of each insertion region (indicating the relative amount of the overexpressed strain) divided by that of *parI* (indicating the relative amount of both the overexpressed and vector control strains). In addition, the relative ratio of the overexpressed strain at each point relative to the initial point was calculated.

### Colony Size Measurement

Each strain was cultivated in LB for 14–16 h and the resultant cultures were washed with LB. Then, the initial OD_600_ of washed cell suspensions was adjusted to 0.03 with LB, and the resultant cell suspensions were serially diluted with CF buffer. Each diluted cell suspension (10 μl) was plated on LB agar and incubated for 48 h at 30°C. To measure the diameters of single colonies, each plate was scanned at a resolution >300 dots per inch and the images were analyzed manually with ImageJ software. The colony size was quantified, and histograms were drawn, using R software (ver. 2.7.0; R Development Core Team, Vienna, Austria).

### Evaluation of the Conjugation Frequency During a Competition Assay

Each RP4-harboring strain [ancestral KT2440(RP4) and evolved 1-L and 2-L strains] (Km^*r*^ by RP4 carriage) and RP4-free KT2440RG strain (Rif^*r*^ and Gm^*r*^) were used as the donor and recipient, respectively. A competition assay was performed using the donor and recipient cells, as described above. In this case, only the first 24-h competitive cultivation with no transfer was performed. After the 24-h cultivation, small portions of the culture were plated on LB agar supplemented with Km, and on LB agar supplemented with Km, Rif, and Gm. Colonies on LB agar supplemented with Km, Rif, and Gm were regarded as transconjugants [KT2440RG(RP4)]. Although transconjugants can also grow on LB supplemented with Km, their proliferation is lower than that of donors and recipients. Therefore, colonies grown on LB supplemented with Km were regarded as donors (RP4-harboring strain). Conjugation frequency was calculated as follows: the number of CFU/ml of transconjugants divided by the number of CFU/ml of donors.

### Whole-Genome Sequencing

Sequencing libraries of KT2440 derivatives were prepared using the Nextera XT DNA Sample Prep Kit (Illumina, San Diego, CA, United States) following the manufacturer’s instructions. Paired-end sequencing of the library ends (2 × 150 bp) was done using the MiSeq platform (Illumina) and a 300-cycle V2 chemistry cartridge (Illumina). Adapter trimming of the obtained reads was done automatically using FASTQ file generation; quality trimming was done using CLC Genomics Workbench (ver. 6.0; CLC Bio, Aarhus, Denmark) with the default parameters. Trimmed reads were mapped to the genome sequence of KT2440 (GenBank accession number AE015451) and RP4 (GenBank accession number L27758) using the Map Reads to Reference function of CLC Genomics Workbench with the default parameters; the mapping results were used for variant calling and analysis of genomic rearrangements.

### RNA Extraction

Total RNA extraction from the *Pseudomonas* strain was performed as described previously (Takahashi et al., 2015). Each strain was cultivated in SUC medium and the culture was treated with the same volume of RNAprotect Bacteria Reagent (Qiagen, Hilden, Germany) to stabilize RNA at each sampling point. Total RNA was extracted using NucleoSpin RNA II (Macherey-Nagel, Düren, Germany). After the eluted RNA was treated with RQ1 RNase-free DNase (Promega, Madison, WI, United States), the purification was performed again using the same extraction kit.

### 5′ Rapid Amplification of cDNA Ends (5′-RACE) Analysis

To determine the transcriptional start sites (TSSs) of the novel transcription unit in the intergenic region, 5′-RACE analysis was performed. Total RNA of the ancestral KT2440(RP4) strain was extracted; the sampling point was 4 h (log phase) after inoculation. cDNA synthesis and 5′-RACE PCR were performed using the SMARTer RACE cDNA Amplification Kit (Clontech, Mountain View, CA, United States) in accordance with the manufacturer’s instructions. A specific primer (8599-RACE-GSP) designed to anneal the intergenic region between PP_5401 and PP_5402 was used for 5′-RACE PCR. 5′-RACE PCR products were separated by agarose gel electrophoresis and purified. Then, the resultant products were ligated into the pT7BlueT-vector to form pTR8599, and the nucleotide sequences of the amplified DNA fragments were confirmed by Sanger sequencing (FASMAC, Kanagawa, Japan) using the R-20mer primer.

### Quantitative Reverse-Transcription (qRT)-PCR

To evaluate the transcriptional level of the intergenic region, qRT-PCR was performed. Primers in the intergenic region (PP_5401.2-F, R) were designed using the Primer3 program (Rozen and Skaletsky, 2000) (Table 2). Total RNA extraction from KT2440, and from ancestral and evolved KT2440(RP4) strains, was performed as described above, and the sampling points were 2, 4, 6, 8 h after inoculation. cDNA was quantified using an ABI 7300 real-time PCR system (Applied Biosystems, Foster City, CA, United States), as described previously (Takahashi et al., 2015). The data were normalized using the average transcriptional level of 16S rRNA as the internal standard.

### Construction of Vectors for Overexpression of the Novel Transcriptional Unit

Each target region of the identified TSS, with lengths of 100, 200, 300, or 400 bp, was amplified by PCR using KOD-Plus-Neo (Toyobo, Osaka, Japan) and the primer sets listed in Table 2. Each resultant fragment was cloned into pBBR1MCS-5 (Kovach et al., 1995) using Ligation High (ver. 2; Toyobo), yielding pBBR100, pBBR200, pBBR300, or pBBR400. The resultant vectors were introduced into the KT2440 strain by electroporation.

### RNA-Seq Analysis

For RNA-sequencing (RNA-Seq) analysis, total RNA was extracted from the KT2440(pBBR1MCS-5) [KT(vc)] and KT2440(pBBR400) [KT(400)] strains as described above, except that the RNeasy Mini Kit (Qiagen) was used instead of NucleoSpin RNA II. Two biological replicates of RNA were extracted from each strain grown in SUC medium supplemented with Gm, and the sampling point was 4 h (log phase) after inoculation. To remove rRNA from total bacterial RNA, we used Ribo-Zero rRNA Removal Kits for Gram-negative bacteria (Epicentre, Madison, WI, United States) and checked the quality using the 2100 Bioanalyzer instrument and the RNA 6000 Pico chip (Agilent Technologies, Santa Clara, CA, United States). To construct the library, we used the TruSeq RNA Library Prep Kit v2 (Illumina). The sequencing run was performed using a MiSeq Reagent Kit (150-cycle) and an Illumina Genome Analyzer. FASTQ data generated by MiSeq were imported into CLC Genomic Workbench (ver. 7.0; CLC Bio) and quality checks and trimming were performed. Reads were mapped to reference sequences (KT2440: GenBank accession No. AE015451, pBBR1MCS-5: GenBank accession No. U25061) and the reads per kilobase per million mapped reads (RPKM) value for each open reading frame (ORF) was calculated using CLC Genomic Workbench. All of the biological duplicate data were included in the analysis, and the RPKM values for each ORF showed a correlation >0.97 between the duplicate samples (Supplementary Figure S1). We identified upregulated and downregulated ORFs with RPKM fold-change values >2.0 in all four comparisons [i.e., of replicates 1 and 2 of the KT(vc) sample and replicates 1 and 2 of the KT(400) sample].

### Data Deposition

The raw sequence data of this study have been deposited in the Sequence Read Archive^1^ under project number PRJNA611485 (whole-genome sequencing) and PRJNA602577 (RNA-Seq analysis).

### Statistical Analyses

The conjugation frequency of RP4 and expression level of the sRNA region were analyzed using the F-test and Student’s *t*-test (*P* < 0.05). The colony size was compared among ancestral and isolated strains using one-way analysis of variance followed by Tukey’s *post hoc* test (*P* < 0.05).

## Results

### Rapid Amelioration of the Fitness Cost Imposed by RP4 Carriage on KT2440

**FIGURE 1:**
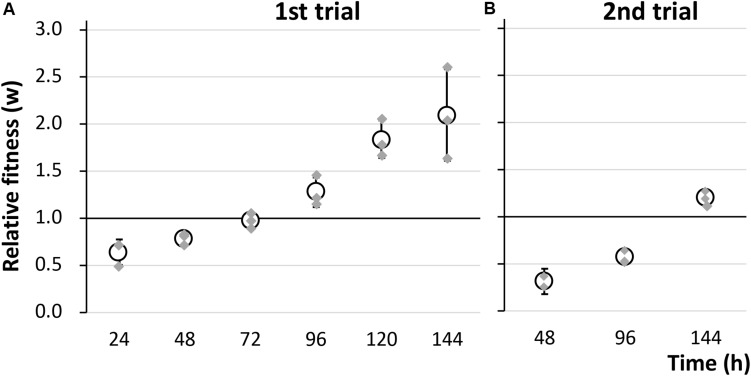
The fitness of strain KT2440(RP4) was evaluated by a competition assay using two biological replicates. The relative fitness (W) was calculated **(A)** every 24 h in the first trial and **(B)** every 48 h in the second trial. Means and standard deviations (error bars) of triplicate data (shown by gray diamonds) are shown.

**FIGURE 2:**
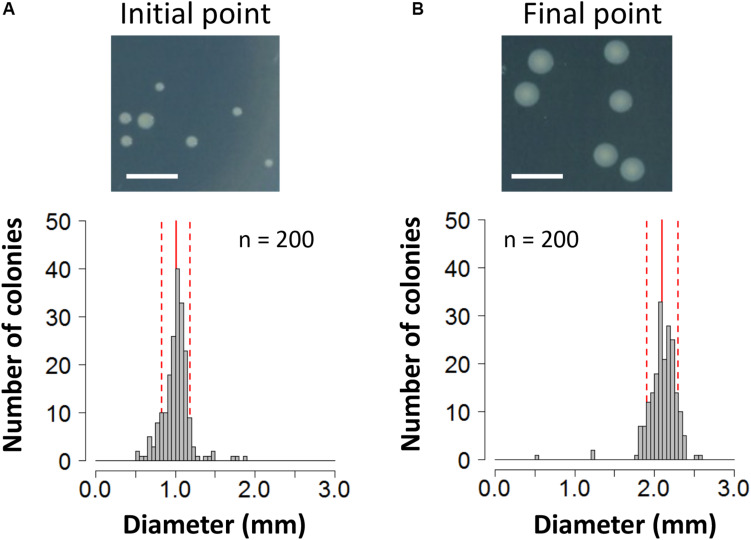
Photographs and histogram of the colony diameter sizes of the KT2440(RP4) strain at **(A)** the start point of the competition assay and **(B)** the end point of the 144-h assay. For each strain, colonies were cultivated on L broth agar plates supplemented with Km at 30°C for 48 h. Scale bar = 5 mm. The diameters of 200 colonies were measured. Means and standard deviations are shown by red solid and broken lines, respectively.

To evaluate the fitness cost imposed by plasmid RP4 on KT2440 cells, the survival of KT2440(RP4) cells in a mixed culture with RP4-free KT2440 cells was assessed using the W value (Lenski et al., 1991) as an index for fitness. After the first 24-h competitive cultivation, the W value was 0.64 ± 0.13 (Figure 1A), indicating that the fitness of KT2440(RP4) was decreased by RP4 carriage. Unexpectedly, the W value then gradually increased, reaching 2.10 ± 0.49 at the final point of the assay, suggesting that the fitness of KT2440(RP4) improved rapidly during cultivation (Figure 1A). In the second trial, in which the W value was calculated every 48 h, the value increased similarly and reached 1.20 ± 0.08 at the final time point (Figure 1B). We confirmed that KT2440(RP4) showed a slightly slower growth rate than that of RP4-free KT2440 (Supplementary Figure S2). In addition, the plasmid persistence of RP4 was assessed by pure culture of KT2440(RP4) in SUC medium for 24 h with shaking. The ratio of the RP4-harboring strain was maintained at 100% after the 24-h cultivation, indicating that RP4 was stable in KT2440. Based on these results, the fitness cost imposed by RP4 carriage was ameliorated by adaptive evolution of this strain. Interestingly, the W value exceeded 1.0 at the end of the experiment, indicating that KT2440(RP4) could eventually dominate in the competitive culture. In addition, we observed a morphological change in colonies of the RP4-harboring strain. At the beginning of the competition assay, the mean colony diameter was 1.01 ± 0.17 mm and >98% of colonies were smaller than 1.5 mm (Figure 2A). After evolving, the mean colony diameter increased to 2.10 ± 0.19 mm and >98% of colonies were larger than 1.5 mm (Figure 2B). The increased colony size might be due to the change in fitness, but the fact that KT2440(RP4) also acquired a novel phenotype of large colony size (similar to the size of the plasmid-free ancestral strain) via adaptive evolution is intriguing.

**FIGURE 3:**
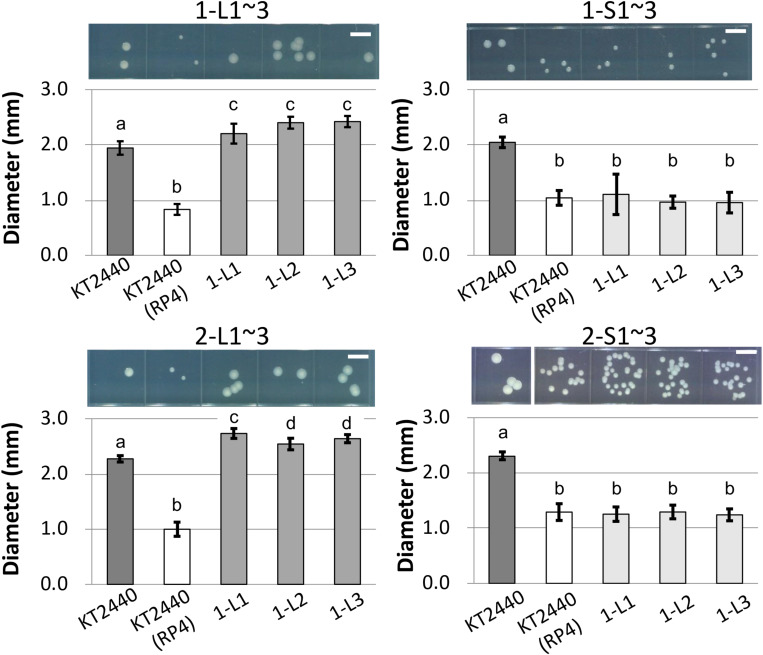
Photographs and measurements of colony diameters of ancestral RP4-harboring and RP4-free KT2440, of and 1-L, 1-S, 2-L, and 2-S strains. For each strain, colonies were cultivated on L broth (LB) agar plates at 30°C for 48 h. Scale bar = 5 mm. The diameters of 50 colonies were measured. Means and standard deviations (error bars) are shown. The lowercase letters a, b, c, and d in each figure indicate significant differences (one-way analysis of variance with Tukey’s *post hoc* test; *P* < 0.05; *n* = 50).

**FIGURE 4:**
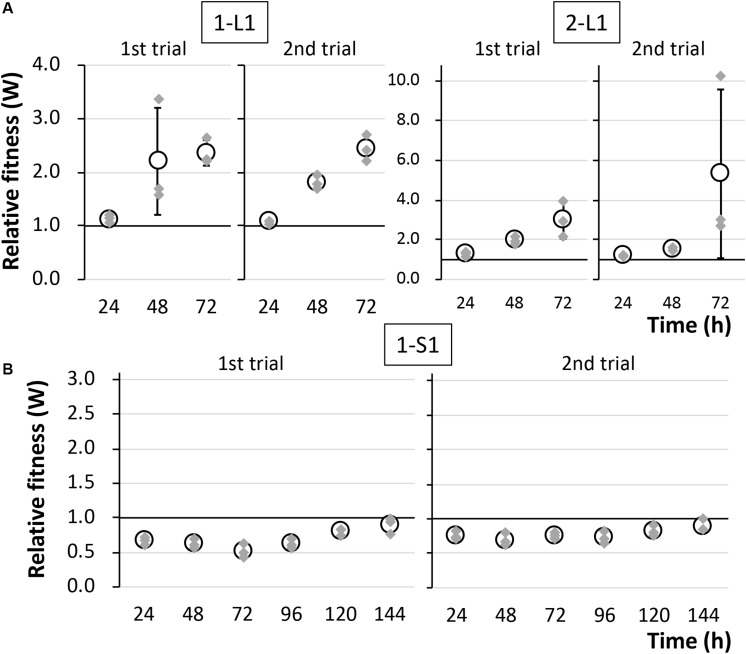
The fitness of strains **(A)** 1-L1 and 2-L1 and **(B)** 1-S1 was evaluated by competition assays using two biological replicates. The relative fitness (W) was calculated every 24 h. Means and standard deviations (error bars) of triplicate data (indicated by the gray diamonds) are shown.

To determine the reason for the reduced fitness cost, three large colonies were isolated from each of the two independent competitive cultures; the strains were named 1-L1–3 and 2-L1–3 (Table 1). As control strains, small colonies were similarly isolated and named 1-S1–3 and 2-S1–3 (Table 1). We confirmed the colony size of each isolated strain, and the trends therein, by comparison with the ancestral strain, as shown in Figure 3 and Supplementary Figure S3. Next, the fitness of the isolated strains was assessed; the results for several selected strains are shown in Figure 4. As expected, the competition assay indicated that the W values of strains 1-L1 and 2-L1 did not decrease and exceeded 1.0 (Figure 4A). Meanwhile, the W value of 1-S1 decreased initially but then slightly increased relative to that of the ancestral KT2440(RP4) strain (Figure 4B). These results suggested that RP4 carriage did not impose any fitness cost on 1-L1 or 2-L1, but it did impose a cost on 1-S1. Moreover, the 1-L and 2-L strains showed a slightly higher growth rate compared with the ancestral, 1-S, and 2-S strains (Supplementary Figure S4). To assess the impact of conjugation of RP4 from RP4-harboring strains (ancestral strains and evolved strains 1-L1 and 2-L1) to RP4-free strains during the competition assay, the conjugation frequency during 24-h competitive cultivation was evaluated. Very low conjugation frequencies were detected (Supplementary Figure S5), and there was no significant difference in the conjugation frequency of RP4 between the ancestral strain and each evolved strain (Student’s *t*-test; *P* > 0.05). Given the very low ratio of transconjugants after 24-h competitive cultivation (<0.012%), the impact of conjugation on the fitness measurement in the competition assay can be regarded as negligible, even after 144 h of cultivation. Based on these results, the isolated 1-L and 2-L strains were defined as “evolved strains,” which show a lower RP4 carriage-imposed fitness cost, larger colony size, and higher growth rate compared with the ancestral strain.

### A Novel Transcriptional Unit Involved in the RP4 Fitness Cost Located on the KT2440 Chromosome

To identify mutations in the genomes of the evolved strains, we performed whole-genome sequencing analyses on 12 isolated strains (1-L1–3, 2-L1–3, 1-S1–3, and 2-S1–3) and the ancestral KT2440(RP4) strain. Although no mutation was found on RP4 in any strain, several mutations were found on their chromosomes. In the 1-S and 2-S strains, no significant mutations were found, and no mutations were found in the 2-S2 genome (Table 3). For evolved strains, a single nucleotide substitution was commonly found at position 6,158,637 of the chromosomes of the 1-L strains (Table 3). Similarly, a single replacement was commonly found at position 6,158,719 on the chromosomes of the 2-L strains (Table 3). Interestingly, these two mutations on evolved strains were located very close together, i.e., in the same intergenic region between PP_5401 and PP_5402. A previous transcriptome analysis suggested that this intergenic region was transcribed in the same direction as PP_5402 in the plasmid-free KT2440 strain at log phase (Supplementary Figure S6) (Takahashi et al., 2015). WebGeSTer DB (a transcriptional terminator database) analysis (Mitra et al., 2011) revealed no terminator region in the intergenic region between PP_5401 and PP_5402. Recently, sRNA transcripts annotated in intergenic regions were identified on the KT2440 chromosome via transcriptional analysis under various stress conditions (Bojanovič et al., 2017). That study reported three novel sRNAs (Pit174, 175, and 176) in our target intergenic region between PP_5401 and PP_5402.

**TABLE 3:** Point mutations identified in isolated strains.

Position	Gene^a^	Product	Identified genetic change or deletion											
			1-S1	1-S2	1-S3	2-S1	2-S2	2-S3	1-L1	1-L2	1-L3	2-L1	2-L2	2-L3
2,061,008	PP_1837/PP_1838	–			C→T									
2,504,321	PP_2198	Glucose sorbosone dehydrogenase						C→T						
3,935,513	PP_3467/PP_3468	–	G→–	G→–										
4,552,435	PP_4039	Transcriptional regulator				G→T								
5,004,299	PP_4410	Hypothetical protein						T→C						
5,672,092	PP_4979	Hypothetical protein												G→C
6,158,637	PP_5401/PP_5402	–							G→C	G→C	G→C			
6,158,719	PP_5401/PP_5402	–										G→A	G→A	G→A

*^*a*^For intragenic mutations, gene names are shown. For intergenic mutations, adjacent ORFs are listed such as A/B.*

**FIGURE 5:**
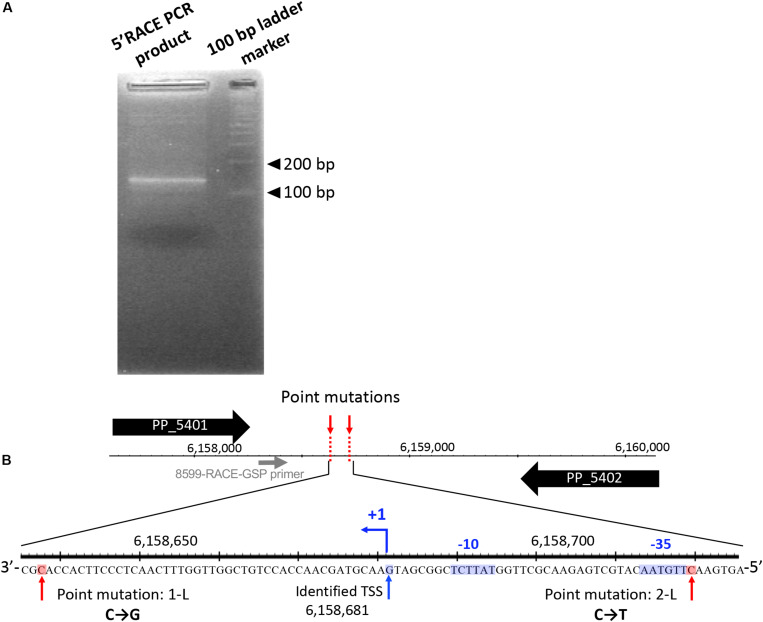
5′ rapid amplification of cDNA ends (5′-RACE) analysis was performed to determine transcriptional start sites (TSSs) in this intergenic region. **(A)** Agarose gel electrophoresis of 5′-RACE polymerase chain reaction (PCR) products. **(B)** The nucleotide sequences determined around TSS are shown. The primer for 5′-RACE PCR (8599-RACE-GSP) is shown by the gray arrow. The blue arrow (+1) indicates the position of the identified TSS. The -35 and -10 elements identified are highlighted in blue, and the two point mutations are highlighted in red.

**FIGURE 6:**
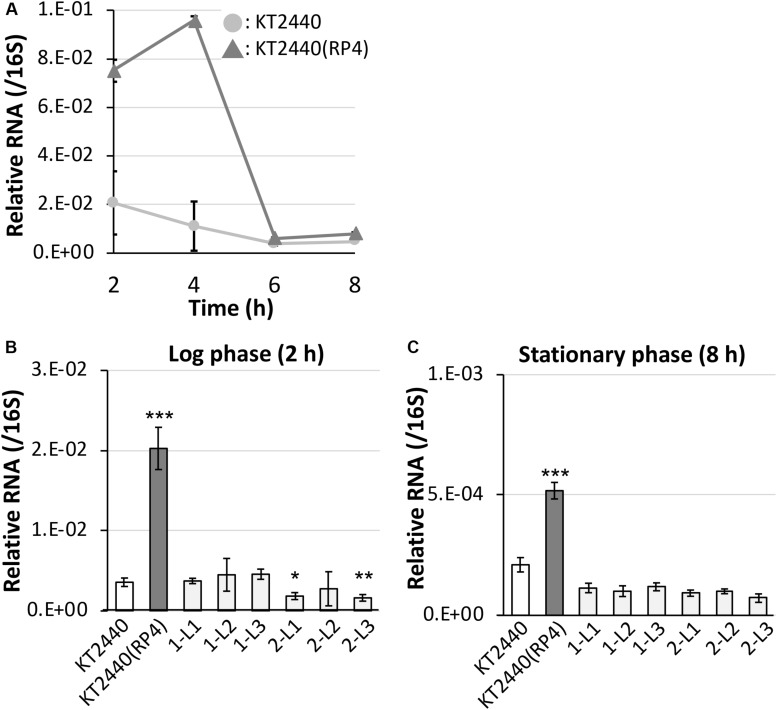
Quantitative reverse-transcription polymerase chain reaction analysis of the small RNA region **(A)** in RP4-free and RP4-harboring KT2440 strains at each time point, and **(B)** among ancestral RP4-free/-harboring KT2440 and evolved strains at log phase and **(C)** stationary phase. All data were normalized using the average transcriptional level of 16S rRNA as the internal standard. Means and standard deviations (error bars) of triplicate data are shown. Asterisks indicate significant differences compared with the results of the KT2440 strain, as assessed by the *F* test and Student’s *t*-test (*P < 0.05, **P < 0.01, ***P < 0.005; *n* = 3).

First, we performed 5′-RACE analysis on total RNA from KT2440(RP4) grown until log phase, to determine the TSSs of this sRNA region. When the 8599-RACE-GSP primer that anneals specifically to the intergenic region was used, the specific PCR product was obtained (Figure 5A). Sequencing of the resultant DNA fragment suggested position 6,158,681 as a TSS of the sRNA, where this position is very close to that of Pit174 (Figure 5B and Supplementary Figure S7). Additionally, putative -10/-35 elements for σ^70^ of pseudomonads (Domínguez-Cuevas and Marqués, 2004) were found upstream of the identified TSS. One mutation (at position 6,158,637) occurred within the sRNA, while the other (at position 6,158,719) was in its promoter region (Figure 5B). Furthermore, qRT-PCR analysis on this intergenic region revealed that RP4 carriage resulted in strongly elevated transcription of this region at log phase (Figure 6A). In addition, we compared the transcriptional level of this region among KT2440, KT2440(RP4), and evolved strains (1-L1–3, 2-L1–3) by qRT-PCR. The transcriptional level in evolved strains was clearly lower than that in the ancestral KT2440(RP4) strain and similar to that in the plasmid-free KT2440 strain at log phase (Figure 6B). At stationary phase, the transcriptional level in all strains was clearly lower than that at log phase, but differences in transcriptional level were observed among all strains and the tendency was similar to that at log phase (Figure 6C). These results clearly show that the transcriptional level of this sRNA between PP_5401 and PP_5402 was increased by RP4 carriage in the ancestral strain, and compensatory mutations in this region could decrease the transcriptional level.

**FIGURE 7:**
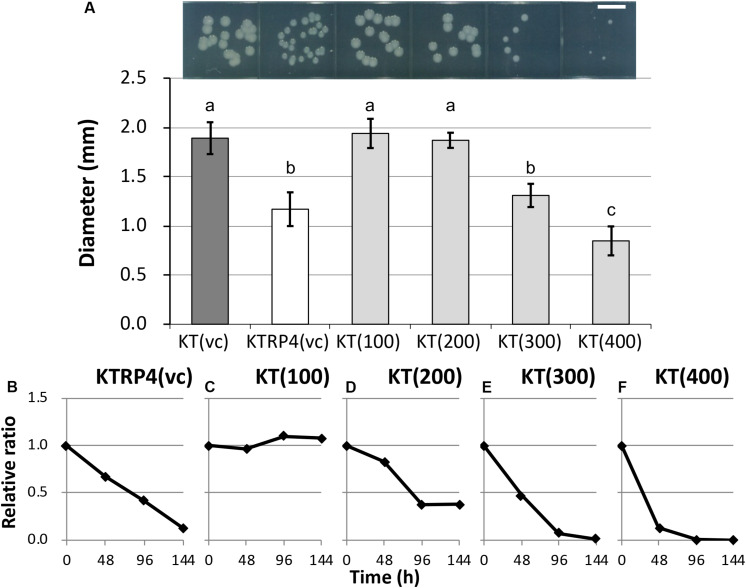
**(A)** Photographs and measurements of colony diameters among control and overexpressed strains. For each strain, colonies were cultivated on L broth agar plates supplemented with Gm at 30°C for 48 h. Scale bar = 5 mm. The diameters of 50 colonies were measured. Means and standard deviations (error bars) are shown. The lowercase letters a, b, and c indicate significant differences (one-way analysis of variance with Tukey’s *post hoc* test; *P* < 0.05; *n* = 50). The fitness of strains **(B)** KTRP4(vc), **(C)** KT(100), **(D)** KT(200), **(E)** KT(300), and **(F)** KT(400) was evaluated by competition assays. The relative ratio, calculated as the proportion of overexpression strains at each time point divided by the initial proportion of overexpression strains, was based on the band intensities of polymerase chain reaction products. The value was calculated every 48 h.

To identify the essential region of the sRNA involved in the fitness cost imposed by RP4, overexpression of 100-, 200-, 300-, and 400-bp regions from the TSS was induced from an artificial vector pBBR1MCS-5 (Kovach et al., 1995) in plasmid-free KT2440 cells. Regarding their colony sizes on plate cultures, overexpression of the 300-bp [KT(300) strain] or 400-bp [KT(400) strain] region resulted in colonies that were clearly smaller than that of the RP4-free control strain [KT(vc) strain], while the impact of overexpression of the 100-bp [KT(100) strain] or 200-bp [KT(200) strain] regions was not significant (Figure 7A and Supplementary Figure S8). This result suggested that the region longer than 300 bp was at least effective for reducing colony size. Next, a competition assay with the KT(vc) strain was performed for each overexpression strain, to evaluate the fitness cost imposed by overexpression of the respective regions. To determine the ratio of the overexpressed strain in the competitive culture, PCR amplification of the DNA regions, including vector inserts, was performed using total DNA extracted from the competitive culture. The resultant band intensities on agarose gels were quantified to obtain the ratio of the overexpressed strain in the culture. The relative ratio at each time point was calculated as follows: the ratio of the overexpressed strain divided by the initial ratio. As the control experiment, KT(vc) and KTRP4(vc) were subjected to a competition assay, and the proportion of KTRP4(vc) was calculated by dividing the band intensity of the *trfA2* gene on RP4 by that of the *parI* gene on the KT2440 chromosome. Consequently, the relative ratio was markedly decreased, suggesting that this PCR-based method could quantify the fitness cost (Figure 7B). In the case of overexpressed strains, PCR amplification of the *parI* gene and the insert region of the pBBR1MCS-5 vector was performed, and the ratio of overexpressed strain was calculated based on the band intensities (ratio between the band intensity for the insert region and that for *parI*). While overexpression of the 100-bp region did not impose a fitness cost on the overexpression vector-harboring host (Figure 7C), overexpression of ≥200 bp of the region clearly imposed a fitness cost (Figures 7D–F). Moreover, the fitness cost was higher when the overexpressed region was longer (Figures 7C–F). When 300- or 400-bp regions were overexpressed, the ratio of the overexpressed strain was drastically decreased, similar to the KTRP4(vc) strain, indicating that a region longer than 300 bp was necessary to impose a fitness cost (Figures 7E,F). The basic local alignment search tool (BLAST) was used to explore the function of this 300- or 400-bp region from the identified TSS. However, BLAST search indicated no homology of this region to any protein in the database. Based on these results, we concluded that expression of at least 300 bp of the chromosomal sRNA is effective for imposing an RP4 fitness cost on KT2440 cells, and that the sRNA identified in this study is effectively the same as Pit174 (Supplementary Figure S7). Interestingly, BLAST search revealed that the Pit174 region was completely conserved in several *P. putida* strains at the DNA sequence level. To understand the mechanism of Pit174 function, it will be helpful to assess whether RP4 can load fitness cost on such *P. putida* strains via expression of Pit174. Meanwhile, it has been reported that the RNA chaperone Hfq is involved in the interaction between sRNAs and their target mRNAs (Vogel and Luisi, 2011). To clarify the function of Pit174, we attempted to quantify the RP4 fitness cost in the *hfq* deletion mutant KT2440Δ*hfq* (Arce-Rodríguez et al., 2015). However, deletion of the *hfq* gene itself altered the host phenotype markedly, and we were unable to assess the impact of Hfq on the fitness cost imposed by RP4 (data not shown).

### Impact of the sRNA Region on the Chromosomal Transcriptional Profile

In many studies, disturbance of the host chromosomal transcriptional profile by plasmid carriage was cited as the main cause of the plasmid fitness cost (Harrison et al., 2015; San Millan et al., 2015; Takahashi et al., 2015). Expression of the chromosomal sRNA may have disrupted the transcriptional profile on the plasmid gene(s), thus imposing a fitness cost on the host cell. However, we showed that the expression of the sRNA region clearly imposed a fitness cost in the plasmid-free KT2440 strain (Figures 7E,F). Therefore, the novel sRNA is likely to directly affect host chromosomal function(s). To understand the impact at the transcriptional level, we performed RNA-Seq analysis of the above-prepared KT(400) and KT(vc) strains. We confirmed that the transcription level of the sRNA in KT(400) was clearly higher than that in KT(vc) by RT-PCR, using PP_5401.2-F and -R primers (Supplementary Figure S9). Transcriptome analysis revealed that 27 ORFs were upregulated, and 6 were downregulated, by overexpression of the 400-bp region of the sRNA (Table 4). The proportion of differentially transcribed ORFs was 0.6%, suggesting that disturbance of the host chromosomal transcriptome was relatively low.

**TABLE 4:** Differentially transcribed ORFs in KT(400) strains.

No.	Gene name	Locus tag	COG^a^	Products	Category*^b^*
1	PP_0042	PP_0042	–	Hypothetical protein	Up
2	PP_0608	PP_0608	–	Hypothetical protein	Up
3	*oprH*	PP_1185	M	Outer membrane protein H1	Up
4	*phoP*	PP_1186	T	Two component transcriptional regulator	Up
5	*lon-1*	PP_1443	O	ATP-dependent protease La	Up
6	PP_1487	PP_1487	S	Hypothetical protein	Up
7	*ibpA*	PP_1982	O	Heat shock protein Hsp20	Up
8	PP_2197	PP_2197	–	Hypothetical protein	Up
9	PP_2198	PP_2198	G	Glucose sorbosone dehydrogenase	Up
10	PP_2285	PP_2285	–	Hypothetical protein	Up
11	*rbsB*	PP_2454	G	Monosaccharide-transporting ATPase	Up
12	PP_2520	PP_2520	–	Hypothetical protein	Up
13	PP_2909	PP_2909	–	Hypothetical protein	Up
14	PP_3172	PP_3172	L	Group II intron-encoding maturase	Up
15	PP_3305	PP_3305	P	TerC family membrane protein	Up
16	PP_3328	PP_3328	R	Ring-cleaving dioxygenase	Up
17	PP_3381	PP_3381	–	ISPpu9, transposase	Up
18	PP_3678	PP_3678	–	Hypothetical protein	Up
19	PP_4025	PP_4025	L	ISPpu15, transposase Orf2	Up
20	PP_4092	PP_4092	L	ISPpu15, transposase Orf1	Up
21	*bkdAA*	PP_4401	C	3-Methyl-2-oxobutanoate dehydrogenase	Up
22	*bkdAB*	PP_4402	C	2-Oxoisovalerate dehydrogenase subunit beta	Up
23	PP_4504	PP_4504	U	Hypothetical protein	Up
24	*grpE*	PP_4728	O	Heat shock protein GrpE	Up
25	*hslV*	PP_5000	O	ATP-dependent protease peptidase subunit	Up
26	*hslU*	PP_5001	O	ATP-dependent protease ATP-binding subunit HslU	Up
27	PP_5403	PP_5403	–	Hypothetical protein	Up
28	PP_0526	PP_0526	L	ISPpu10, transposase	Down
29	PP_0700	PP_0700	P	FecR anti-FecI sigma factor	Down
30	PP_4070	PP_4070	P	Hypothetical protein	Down
31	*exbB*	PP_5306	U	Ferric siderophore transport system protein ExbB	Down
32	*exbD*	PP_5307	U	Biopolymer transport protein ExbD	Down
33	*tonB*	PP_5308	M	TonB family protein	Down

*^*a*^The descriptions for each COG code are as follows: J, translation, ribosomal structure, and biogenesis; A, RNA processing and modification; K, transcription; L, replication, recombination, and repair; B, chromatin structure and dynamics; D, cell cycle control, cell division; Y, nuclear structure; V, defense mechanisms; T, signal transduction mechanisms; M, cell wall/membrane/envelope biogenesis; N, cell motility; Z, cytoskeleton; W, extracellular structures; U, intracellular trafficking, secretion, and vesicular transport; O, posttranslational modification, protein turnover, chaperones; X, mobilome; prophages, transposons; C, energy production and conversion; G, carbohydrate transport and metabolism; E, amino acid transport and metabolism; F, nucleotide transport and metabolism; H, coenzyme transport and metabolism, chromosome partitioning; I, lipid transport and metabolism; P, inorganic ion transport and metabolism; Q, biosynthesis of secondary metabolites, transport, and catabolism; R, general function prediction only; S, function unknown; –, uncategorized. ^*b*^Transcription levels of genes in KT(400) in comparison with those in KT(VC). “Up” indicates that the gene is up-regulated in KT(400), and “Down” indicates that the gene is down-regulated in KT(400).*

Next, differentially transcribed ORFs were classified into 11 (of 23) groups based on Clusters of Orthologous Groups of proteins (COG) classifications (Supplementary Figure S10). As shown in Table 5, the relative numbers of ORFs classified as COG code O (post-translational modification, protein turnover; 5 ORFs) and code L (replication, recombination, and repair; 4 ORFs) among the differentially transcribed genes (33 ORFs) were significantly larger than those of ORFs classified as code O (165 ORFs in the whole genome) and code L (200 ORFs in the whole genome) in the whole genome (5,350 ORFs in the whole genome; *P* < 0.05; Fisher’s exact test). The products of five ORFs in the O class (*lon-I*, *ibpA*, *grpE*, *hslV*, and *hslU*) are types of heat shock protein (HSP). Generally, genes encoding HSPs are upregulated in response to various environmental stresses (Lindquist and Craig, 1988; Rohrwild et al., 1997; Teter et al., 1999). In category L, there are several ORFs encoding transposases, but the role of these ORFs in plasmid fitness cost is still unknown. In a *Lactobacillus* strain, some transposase-encoding genes were upregulated in response to various stresses, such as heat stress and acid stress (Palud et al., 2018). Based on these insights, it is predicted that RP4 carriage leads to stressful conditions for KT2440 host cells, where intracellular stress may underlie the fitness cost imposed by RP4.

**TABLE 5:** Numbers of differentially transcribed ORFs in KT(400) strains and their COG categories.

	COG classification^a^																							

COG code	Information storage and processing					Cellular processes and signaling						Metabolism						Un-characterized						
	J	A	K	L	B	D	V	T	M	N	U	O	C	G	E	F	H	I	P	Q	R	S	–	Total
**KT(400) strains**	0	0	0	4^*b*^	0	0	0	1	2	0	3^*b*^	5^*b*^	2	2	0	0	0	0	3	0	1	1	9	33
**Whole genome**	164	2	370	200	2	38	61	232	253	120	37	165	274	200	480	87	163	178	257	79	464	420	1104	5350

*^*a*^The descriptions for each COG code are as follows: J, translation, ribosomal structure, and biogenesis; A, RNA processing and modification; K, transcription; L, replication, recombination, and repair; B, chromatin structure and dynamics; D, cell cycle control, cell division, chromosome partitioning; Y, nuclear structure; V, defense mechanisms; T, signal transduction mechanisms; M, cell wall/membrane/envelope biogenesis; N, cell motility; Z, cytoskeleton; W, extracellular structures; U, intracellular trafficking, secretion, and vesicular transport; O, posttranslational modification, protein turnover, chaperones; X, mobilome; prophages, transposons; C, energy production and conversion; G, carbohydrate transport and metabolism; E, amino acid transport and metabolism; F, nucleotide transport and metabolism; H, coenzyme transport and metabolism; I, lipid transport and metabolism; P, inorganic ion transport and metabolism; Q, biosynthesis of secondary metabolites, transport, and catabolism; R, general function prediction only; S, function unknown; –, uncategorized. ^*b*^The relative number of ORFs classified as a given COG code in the differentially transcribed genes was significantly larger than that of ORFs classified as the same COG code among whole-genome ORFs (*P* < 0.05, Fisher’s exact test).*

Fisher’s exact test also showed that the relative number of ORFs classified as COG code U (intracellular trafficking, secretion, and vesicular transport; 3 ORFs), and containing *exbB* and *exbD*, in the differentially transcribed genes (33 ORFs) was also statistically significantly larger than that of ORFs categorized in COG code U (37 ORFs in the whole genome) in the whole genome ORFs (5,350 ORFs in the whole genome) (*P* < 0.05). Three neighboring ORFs, *exbB*, *exbD* (COG code U), and *tonB* (COG code M), were all downregulated in the sRNA-overexpressed strain. In gram-negative bacteria, TonB-ExbB-ExbD complex is involved in the transport of ferric citrate (Braun, 1995). A domain search suggested that the product of PP_0700 (COG code P), which was a downregulated ORF in the sRNA-overexpressed strain, is a FecR family protein. FecR is the transcriptional regulator of *fecABCDE* genes, which are involved in ferric citrate transport (Angerer et al., 1995; Welz and Braun, 1998). Considering these results, overexpression of the sRNA region induced the downregulation of genes involved in iron acquisition machinery. In a previous study, we reported the common effects of pCAR1 carriage on the iron acquisition system in three *Pseudomonas* hosts (*P. aeruginosa* PAO1, *P. fluorescens* Pf0-1, and *P. putida* KT2440) (Shintani et al., 2010). Fur (ferric uptake regulator) is a transcriptional regulator involved in iron uptake and homeostasis (Thompson et al., 2002) a compensatory mutation in the *fur* gene occurred in plasmid pBP136-harboring *S. oneidensis* MR-1 strains during adaptive evolution (Stalder et al., 2017). Disturbance of the transcriptional regulatory network in the iron metabolism system may underlie the fitness cost imposed by plasmid carriage. Based on these considerations, we suggested that RP4 carriage altered the transcriptional regulatory network involved in the iron metabolism and stress resistance of the KT2440 host cell, resulting in decreased fitness.

Overexpression of the 400-bp sRNA region also upregulated the expression of *oprH* (COG code M), encoding an outer membrane protein, and *phoP* (COG code T), encoding a two-component response regulator. In *P. aeruginosa* PAO1, *oprH* is regulated by the PhoP/PhoQ two-component regulatory system (Macfarlane et al., 1999). Interestingly, it was reported that OprH in KT2440 played a role in controlling the conjugation frequency of the IncP-7 group plasmid pCAR1 (Sakuda et al., 2018). In future work, it will be necessary to explore the molecular function of OprH, to generate interactions between chromosome and plasmids. Furthermore, *bkdAA* and *bkdAB*, which encode branched-chain α-keto acid dehydrogenase, are also upregulated by sRNA overexpression. Two neighboring ORFs are involved in the catabolic pathway of several amino acids, such as lysine, isoleucine, valine, and leucine (Muramatsu et al., 2005; Förster-Fromme and Jendrossek, 2008). Thus, disturbance of amino acid metabolism may be induced by overexpression of the sRNA region.

## Discussion

Generally, plasmid carriage imposes a fitness cost on the host, which can acquire compensatory mutations to reduce the cost (Baltrus, 2013; San Millan and MacLean, 2017; Caroll and Wong, 2018; MacLean and San Millan, 2019). In this study, we revealed that RP4 carriage imposed a fitness cost on KT2440, and that KT2440 could reduce the cost via adaptive evolution. Notably, the evolution in the KT2440 chromosome was very rapid, and was accompanied by alteration of the colony size. The mercury-resistance plasmid pQBR55 imposed a fitness cost on *P. fluorescens* SBW25, but the cost was ameliorated by extremely rapid evolution of the host (Hall et al., 2019). Interestingly, Hall et al. (2019) also reported that this evolutionally pattern was accompanied by alteration of the colony size, as in the case of KT2440(RP4). Small colony variants are frequently found among a wide range of bacteria, including Gram-negative bacteria (Proctor et al., 2006). This morphotype is involved in several phenotypes, such as biofilm formation, antibiotic resistance, and cell fitness (Häußler et al., 2003; Malone, 2015). However, the relationship between plasmid fitness cost and colony size remains unclear, although it is assumed that the colony size becomes small due to the impact of plasmid carriage on the central metabolism. The increase in plasmid copy number also induces a delay in host growth (Engberg et al., 1975; Seo and Bailey, 1985). We calculated RP4 copy numbers in ancestral and evolved strains based on read numbers and coverage of whole-genome sequences. Copy numbers in evolved strains (1.6–2.4) were lower than that in ancestral strain (3.1) and 1-S/2-S strains (3.0–3.7) (Table 6). Although the reason for the decrease in plasmid copy number is still unknown, it may partly explain the improvement in fitness.

**TABLE 6:** Plasmid copy number based on whole genome sequencing.

Strains	Number of total reads	Number of reads on chromosome	Number of reads on plasmid	Coverage of chromosome	Coverage of plasmid	Copy number^a^
KT2440(RP4)	814,192	787,096	23,413	38.2	116.9	3.1
1-L1	2,381,592	2,332,342	35,673	113.2	178.1	1.6
1-L2	2,913,274	2,842,868	55,932	138.0	279.2	2.0
1-L3	2,496,020	2,440,928	38,417	118.5	191.8	1.6
2-L1	2,013,740	1,970,058	34,353	95.6	171.5	1.8
2-L2	2,464,816	2,403,860	48,305	116.7	241.1	2.1
2-L3	2,198,728	2,135,990	49,680	103.7	248.0	2.4
1-S1	2,072,027	2,009,011	58,799	26.5	81.3	3.1
1-S2	3,769,764	3,642,167	120,704	56.4	197.9	3.5
1-S3	2,785,120	2,696,425	83,563	39.1	127.3	3.3
2-S1	955,558	919,201	31,970	16.5	61.8	3.7
2-S2	2,862,190	2,760,179	81,144	41.7	126.6	3.0
2-S3	1,311,846	1,248,070	42,166	60.6	210.5	3.5

*^*a*^Coverage of plasmid is divided by coverage of chromosome.*

The KT2440 strain could obtain compensatory mutations in the intergenic region during the competition assay, which ameliorated the fitness cost imposed by RP4 carriage. As reported by Bojanovič et al. (2017) three novel sRNAs (Pit174, 175, and 176) were annotated in the intergenic region between PP_5401 and PP_5402. In particular, the start position of Pit174 was very close to the identified TSS (Supplementary Figure S7). The length of Pit174 is 307 bp, and we demonstrated that the 300-bp or longer DNA region from the TSS played an important role in the fitness costs imposed by RP4 (Figures 7E,F). Based on these results, we conclude that upregulation of the sRNA corresponding to Pit174 (Supplementary Figure S7) by RP4 carriage (Figure 6A) is key to the fitness costs imposed on KT2440 host cells. The molecular mechanisms underlying the upregulation of Pit174 by RP4 carriage remain unclear. To elucidate these mechanisms, it will be necessary to identify the key gene(s) or region(s) in RP4 involved in the upregulation of Pit174 in future work. Overexpression of this chromosomal sRNA imposed a fitness cost on plasmid-free host KT2440 and altered the transcriptional levels of several genes involved in iron metabolism and the stress response (Table 4). Many sRNAs can regulate the expression of various genes at the transcription level (Storz et al., 2011). Additionally, several sRNAs can also modulate the translation of mRNA (Waters and Storz, 2009) and the sRNA identified on the KT2440 chromosome may function at the translational level to affect the fitness of RP4 host cells.

Previously, we showed that alteration of primary cell functions and reduction of stress resistance by IncP-7 group plasmid pCAR1 carriage were related to the fitness cost of the plasmid in KT2440 host cells (Takahashi et al., 2015). However, previous transcriptome analysis showed that the intergenic region between PP_5401 and PP_5402 was not upregulated by pCAR1 carriage (Takahashi et al., 2015). Additionally, we demonstrated adaptive evolution of KT2440(pCAR1) using an experimental setup similar to that of the present study (Kubo et al., in preparation). In that case, the time required for evolution was much longer than that for KT2440(RP4). Compensatory mutations on the host chromosome could be detected, but the mutations that occurred in KT2440(pCAR1) were completely different from those in KT2440(RP4) (Kubo et al., in preparation). These results strongly suggest that completely different compensatory evolution mechanisms occurred in the same host and were dependent on the type of plasmid. The antecedents of fitness costs are considered to be divergent, because adaptive evolution decreases the cost imposed by each type of plasmid carriage. Thus, it is important to evaluate the impact of other types of plasmid carriage on KT2440, and to clarify the evolutionary machinery involved.

When a plasmid is introduced into the host cell, transcriptional, proteomic, and metabolic profiles are altered greatly (San Millan et al., 2018; Vasileva et al., 2018) consequently, a fitness cost is imposed on the host cell by the plasmid. The molecular basis underlying fitness costs is incompletely known, although several important factors have been identified, such as plasmid replication protein (Sota et al., 2010), helicase (Loftie-Eaton et al., 2015, 2017; San Millan et al., 2015) and global transcriptional regulators (Doyle et al., 2007; Harrison et al., 2015; Stalder et al., 2017). Our study clearly shows that host chromosomal sRNA is another key factor in plasmid fitness costs. Bacterial hosts can obtain compensatory mutations in each hot spot for adaptive evolution, resulting in modulation of host cell functions and a decline in plasmid fitness costs. The diversity of the compensatory mutations drives bacterial adaptation to various environmental conditions. Understanding the relationship between plasmid carriage and sRNA will provide new insight into the molecular mechanisms underlying plasmid fitness costs, and the behavior of plasmid-harboring strains.

## Data Availability Statement

The datasets generated for this study can be found in the PRJNA611485 (whole-genome sequencing), PRJNA602577 (RNA-Seq analysis).

## Author Contributions

HN conceived, designed, and supervised the study. DS and NW performed the experiments. HK analyzed the data and drafted the manuscript. HN edited the manuscript. CS-M, YT, and KO coordinated the study and revised the manuscript. All authors have read and approved the final manuscript.

## Conflict of Interest

The authors declare that the research was conducted in the absence of any commercial or financial relationships that could be construed as a potential conflict of interest.

## Footnotes

1 https://www.ncbi.nlm.nih.gov/sra
