# Supplementary material for: A Novel Small RNA on the Pseudomonas putida KT2440 Chromosome Is Involved in the Fitness Cost Imposed by IncP-1 Plasmid RP4
Source: Front Microbiol. 2020 Jun 23;11:1328. doi: 10.3389/fmicb.2020.01328 (PMC7324555; doi:10.3389/fmicb.2020.01328)
Supplement: Supplementary file 1 [file Data_Sheet_1.PDF]

Supplementary Materials

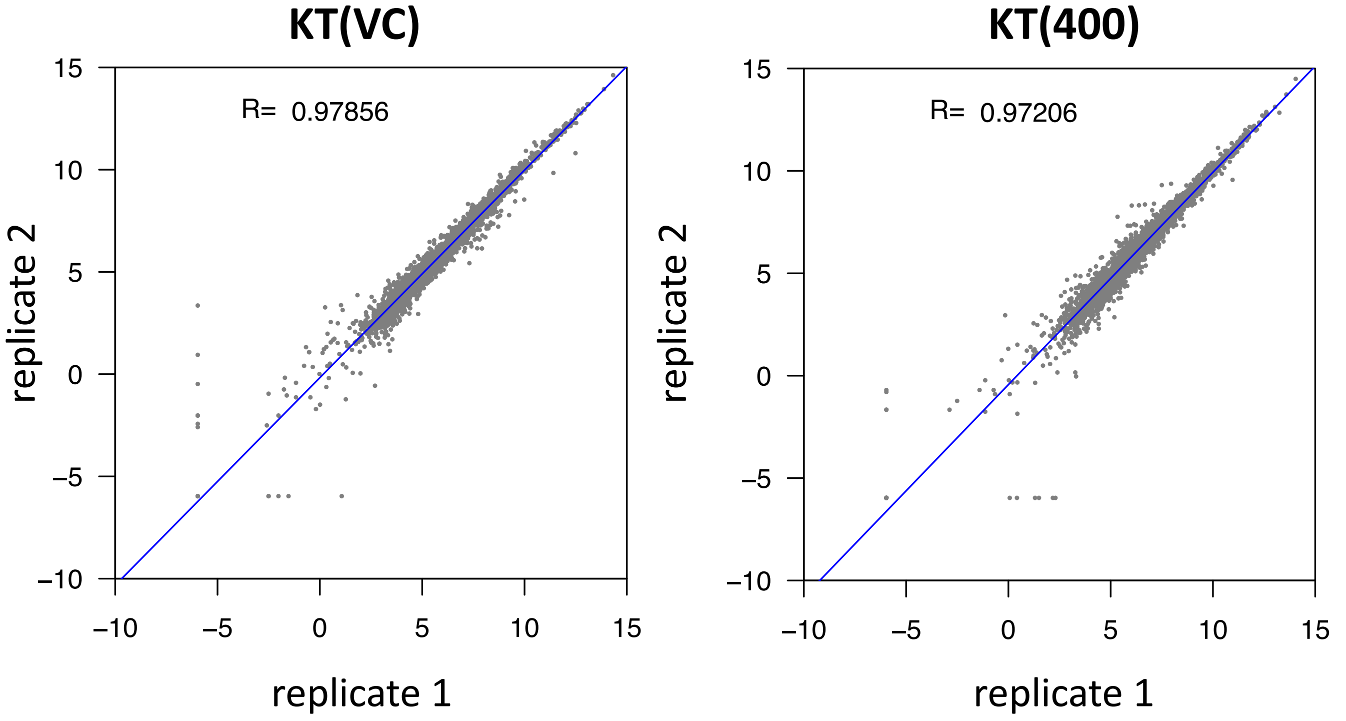

Figure S1. Scatter plots of the reads per kilobase per million mapped reads (RPKM) values of open reading frames (ORFs) on the chromosome. 'R' indicates the correlation coefficient of each plot. The *x*- and *y*-axes are shown in log base 2. Replicates 1 and 2 denote each sample in the two independent samples.

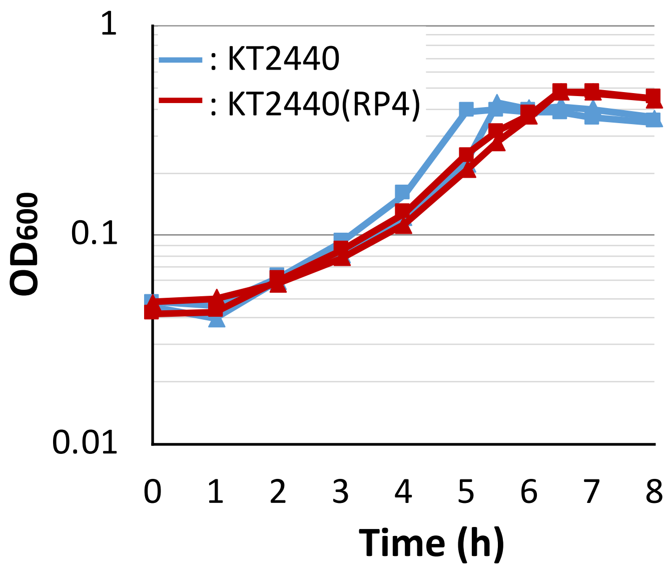

Figure S2. Growth curves of KT2440 and KT2440(RP4) in SUC medium. Means of triplicate data are shown. Squares and triangles represent the results for duplicate experiments.

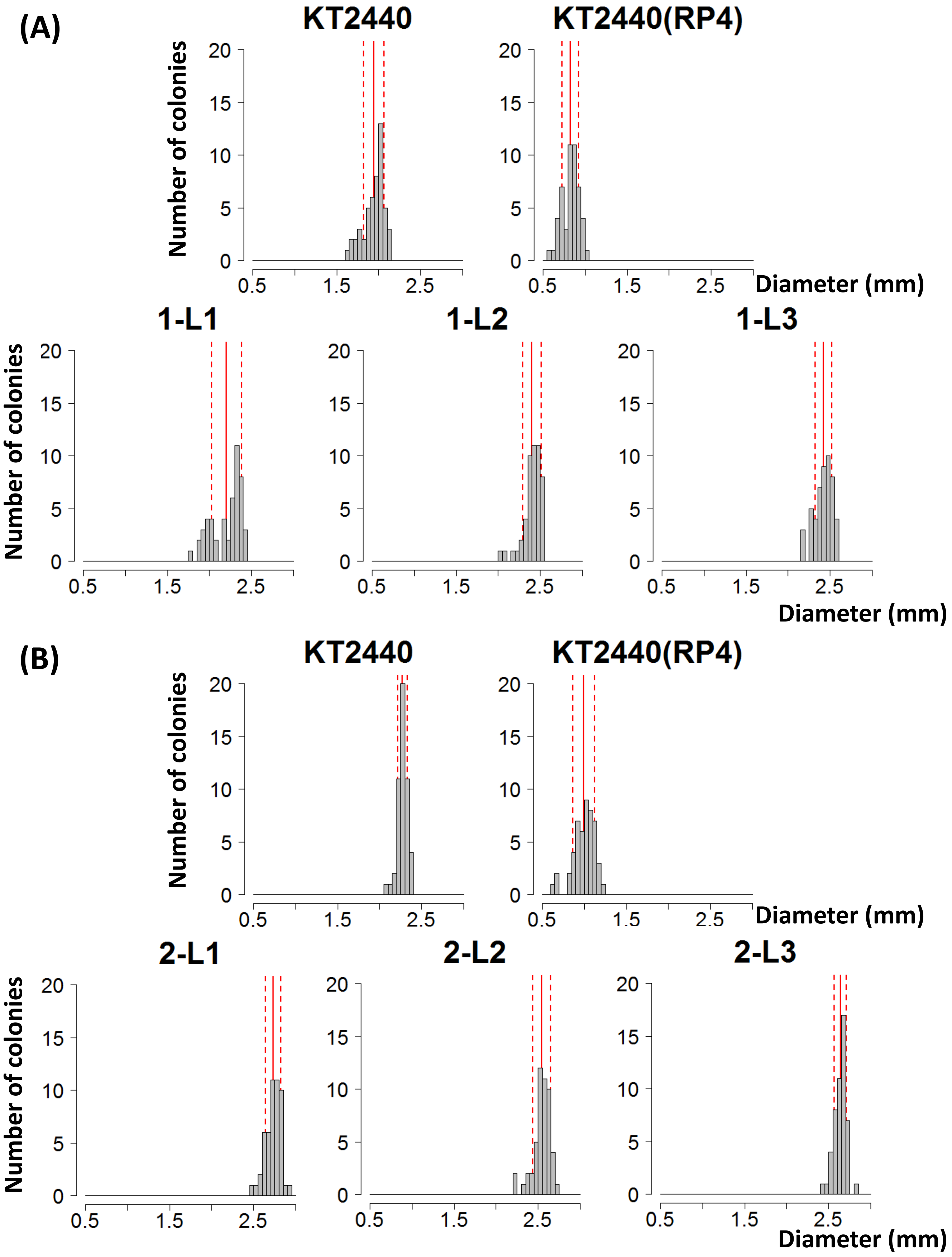

Figure S3. (1/2) Histogram of the colony diameters sizes of ancestral RP4-harboring and RP4-free KT2440, of and (A)1-L, (B) 2-L , (C) 1-S and (D) 2-S strains. For each strain, the diameters of 50 colonies were measured. Means and standard deviations are shown by red solid and broken lines, respectively.

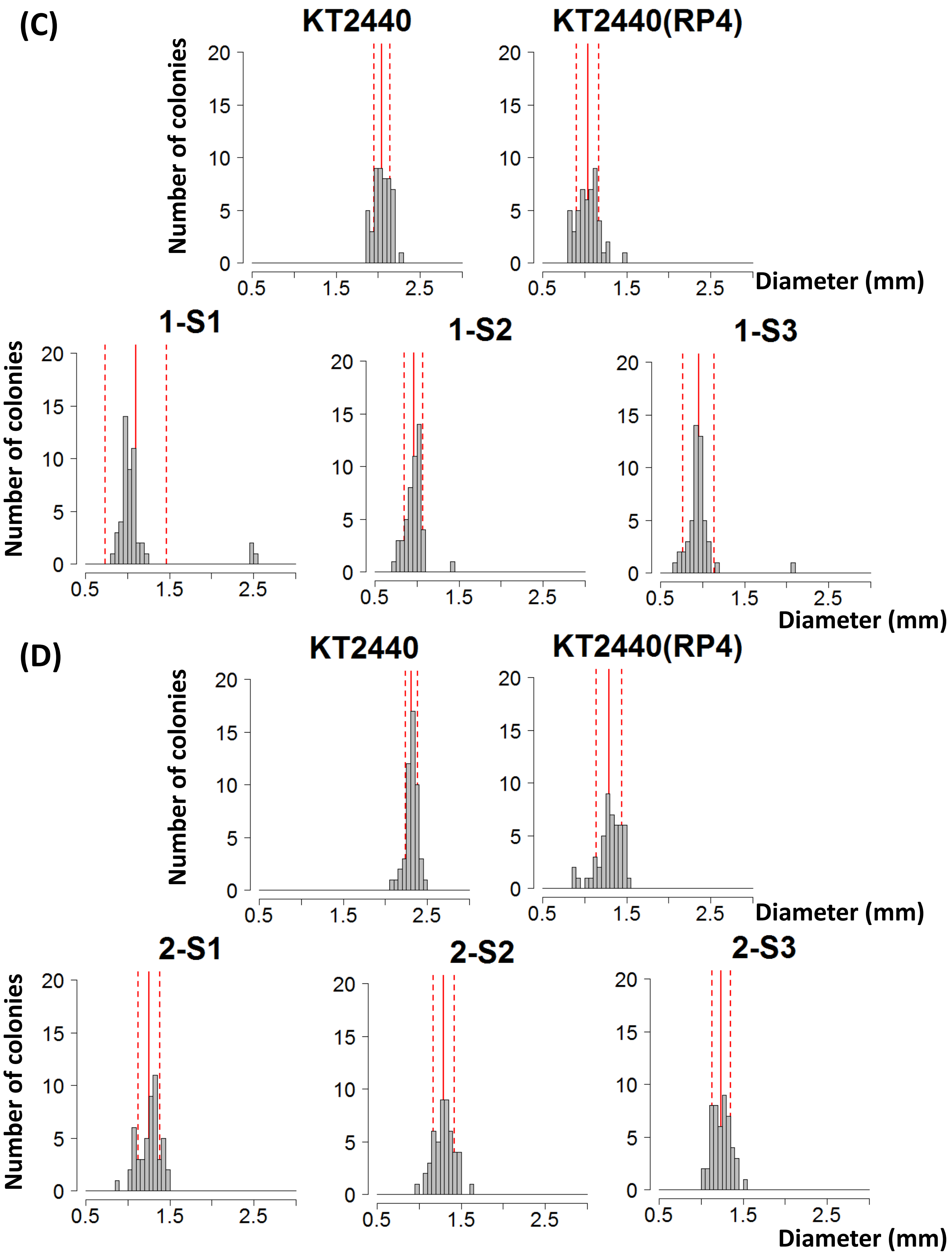

Figure S3. (2/2) Histogram of the colony diameters sizes of ancestral RP4-harboring and RP4-free KT2440, of and (A)1-L, (B) 2-L , (C) 1-S and (D) 2-S strains. For each strain, the diameters of 50 colonies were measured. Means and standard deviations are shown by red solid and broken lines, respectively.

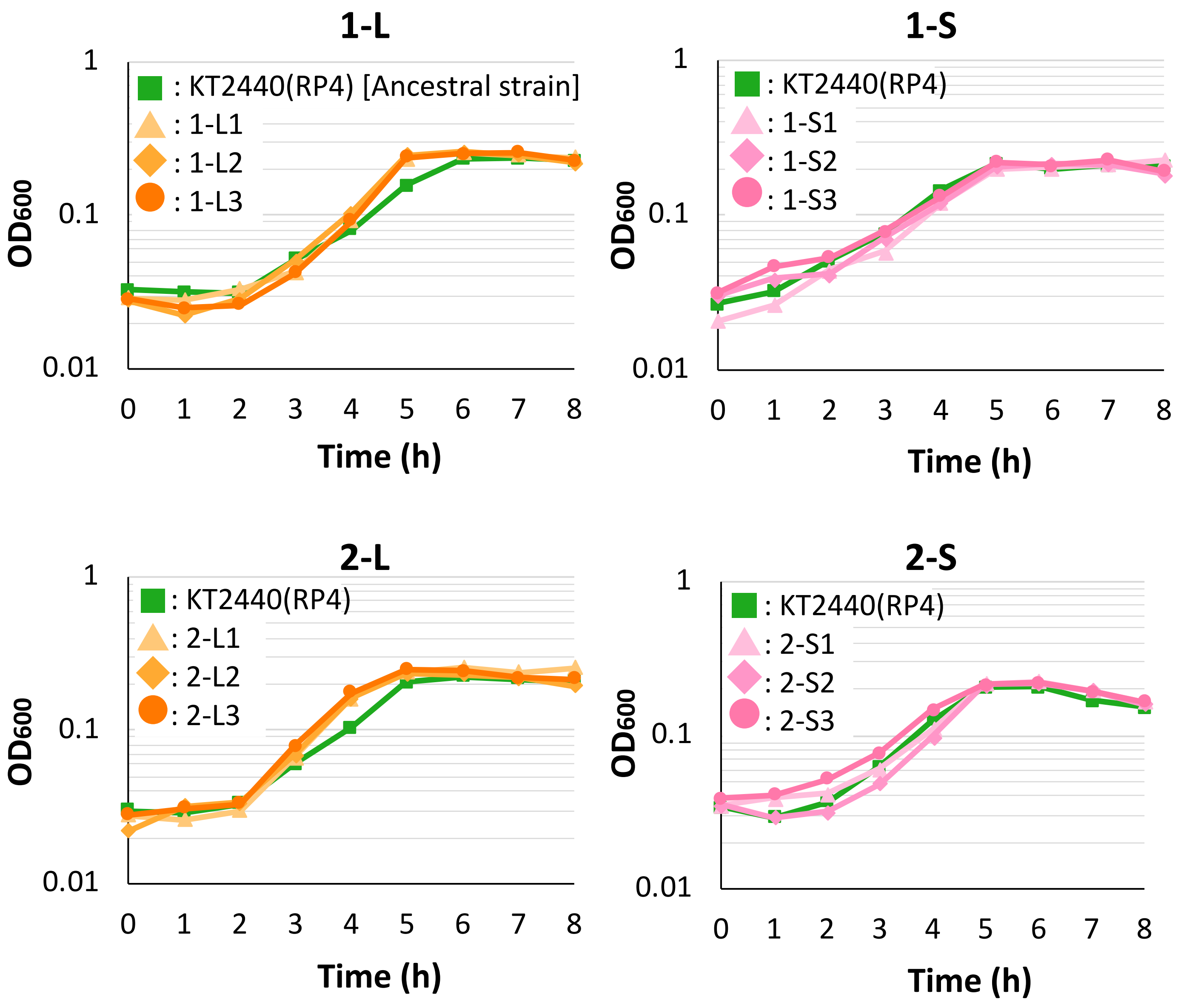

Figure S4. Growth curves of KT2440(RP4) and each isolated strain in SUC medium. Means of triplicate data are shown.

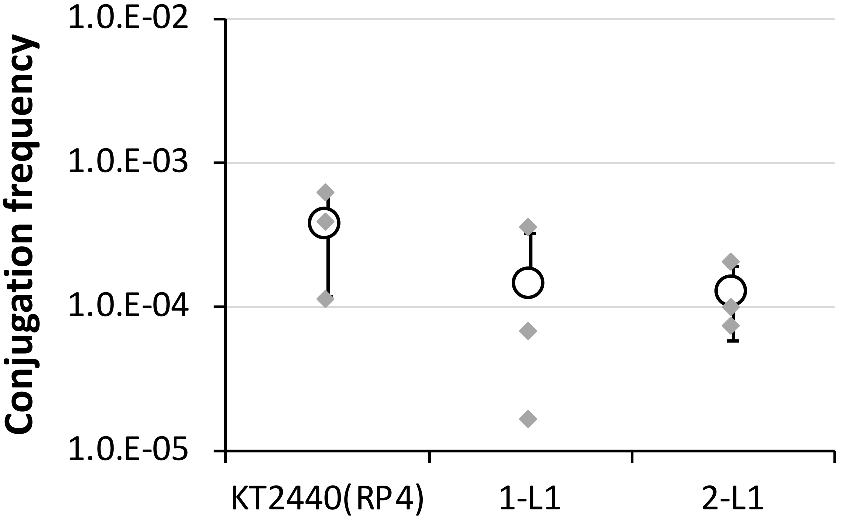

Figure S5. Conjugation frequency of RP4 from each strain to plasmid-free KT2440 strains during 24-hour competitive cultivation. Conjugation frequency was calculated as follows: colony forming units [CFU]/ml of transconjugants divided by CFU/ml of donors. Means and standard deviations (error bars) of triplicate data (shown by grey diamonds) are shown.

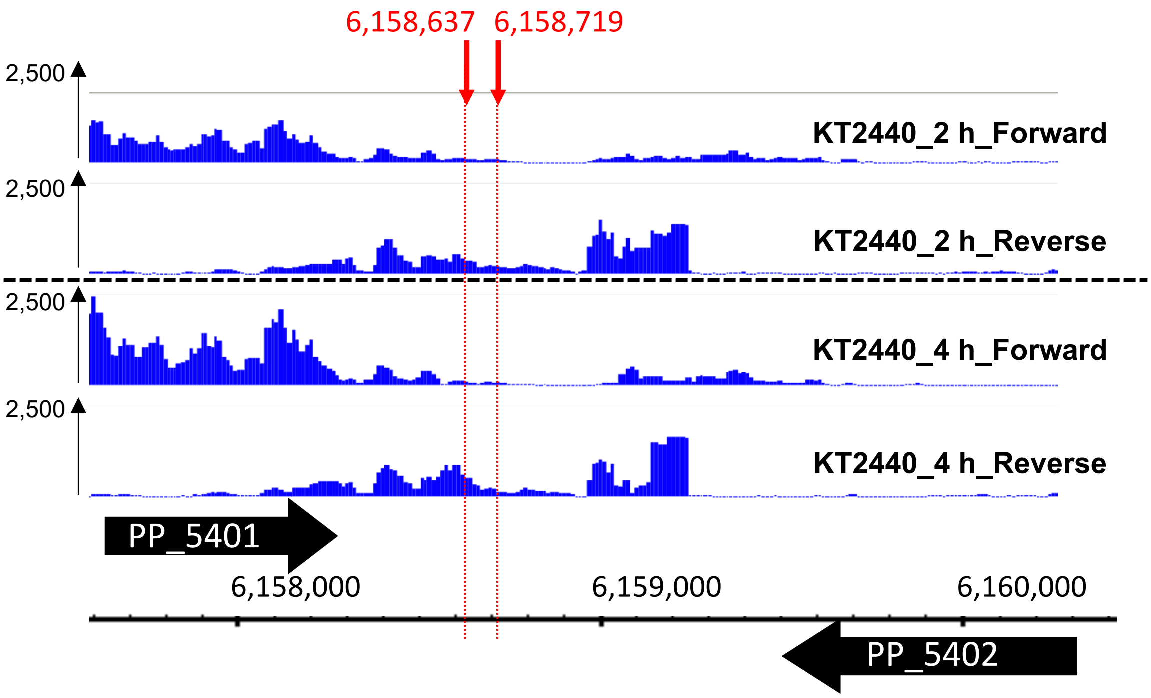

Figure S6. Previous RNA maps around PP_5401 and PP_5402 of 2- and 4-hour-grown plasmid-free KT2440 obtained by tiling array [Takahashi *et al*., 2015]. The x-axis indicates the position on the chromosome and the y-axis indicates the signal intensity. Maps of the forward and reverse strands are shown separately. Identified point mutations are shown on the map (red dotted line).

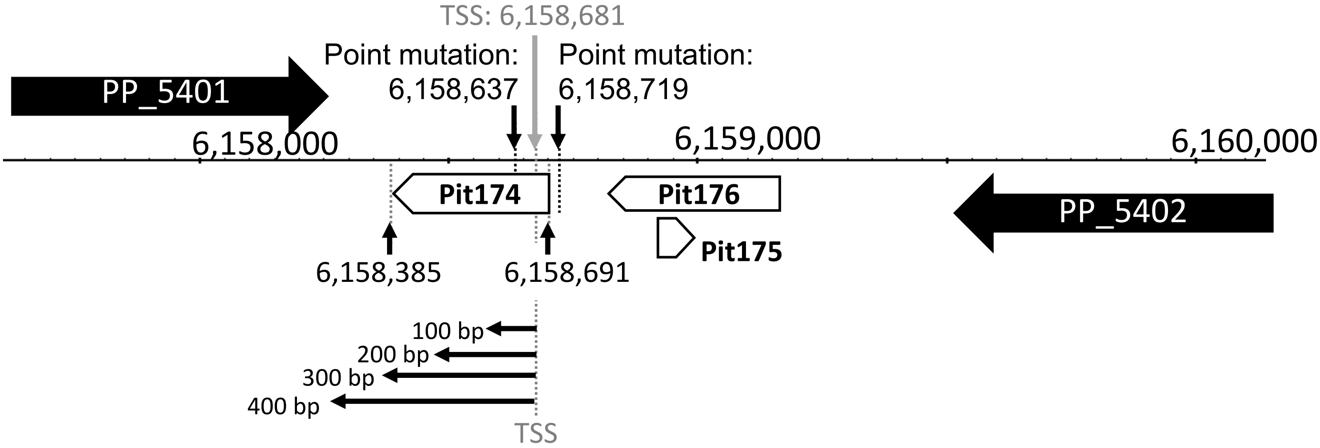

Figure S7. Genetic map of the intergenic region between PP_5401 and PP_5402 on the KT2440 chromosome. The identified transcriptional start site (TSS; position 6,158,681) and two point mutations (positions 6,158,637 and 6,158,719) are shown. Pit174 (from position 6,158,691 to 6,158,385), Pit175, and Pit176 are small RNA (sRNA) regions identified by Bojanovič *et al.* (2017). Arrows indicate the 100-, 200-, 300-, and 400-bp regions from the TSS.

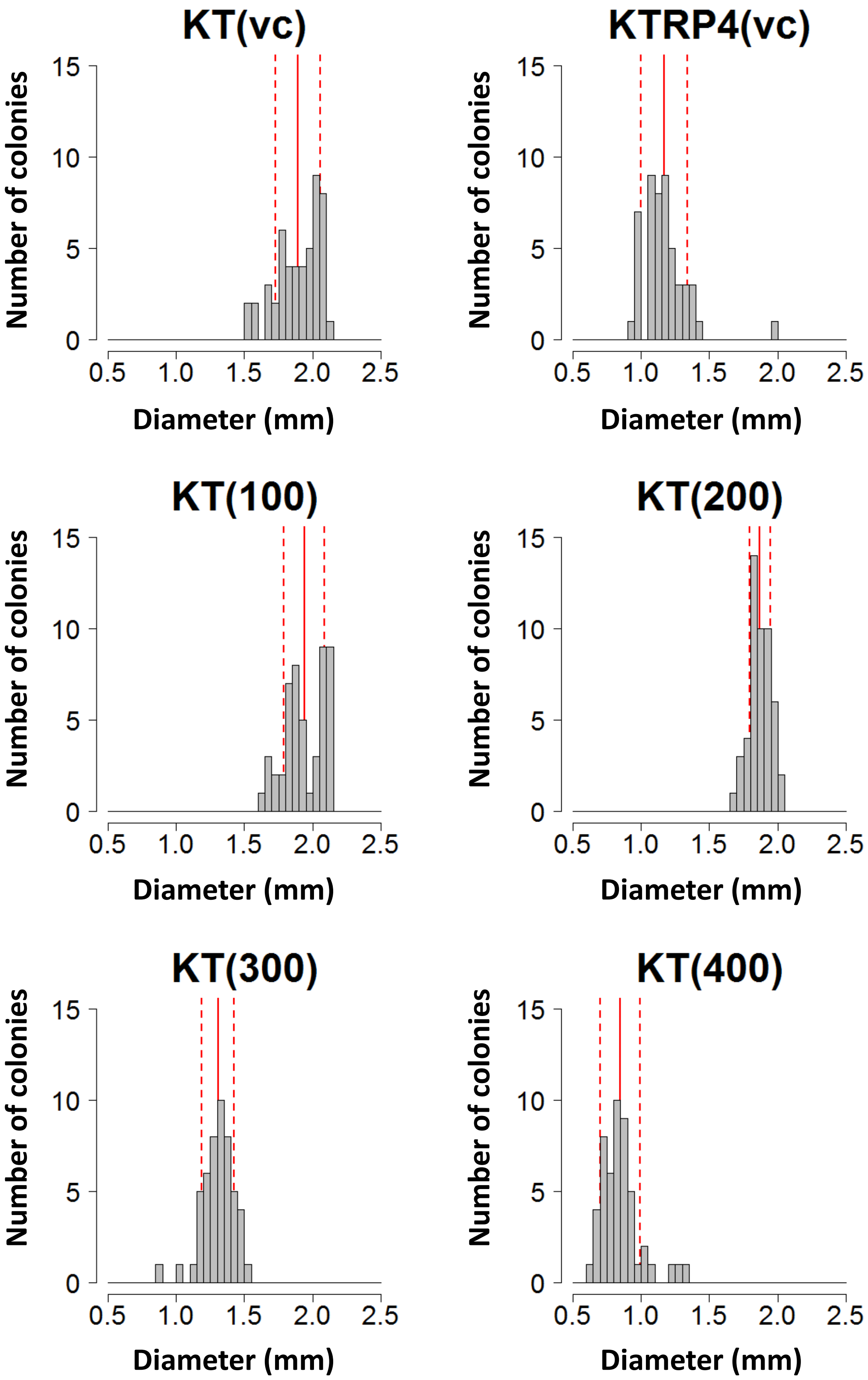

Figure S8. Histogram of the colony diameters sizes of ancestral RP4-harboring and -free KT(vc) strains and each overexpressed strains. For each strain, the diameters of 50 colonies were measured. Means and standard deviations are shown by red solid and broken lines, respectively.

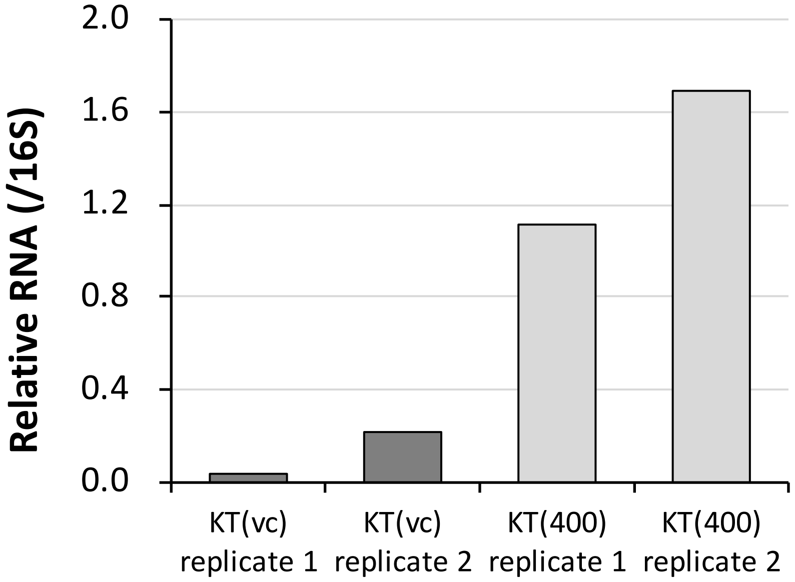

Figure S9. Reverse-transcription polymerase chain reaction (RT-PCR) analysis of the intergenic region in the KT(vc) and KT(400) strains. After agarose gel electrophoresis, the amplified band intensity from each sample was measured using ImageJ software (NIH, Bethesda, MD, USA). In each sample, the relative RNA level was calculated as follows: band intensity of target region divided by that of 16S rRNA.

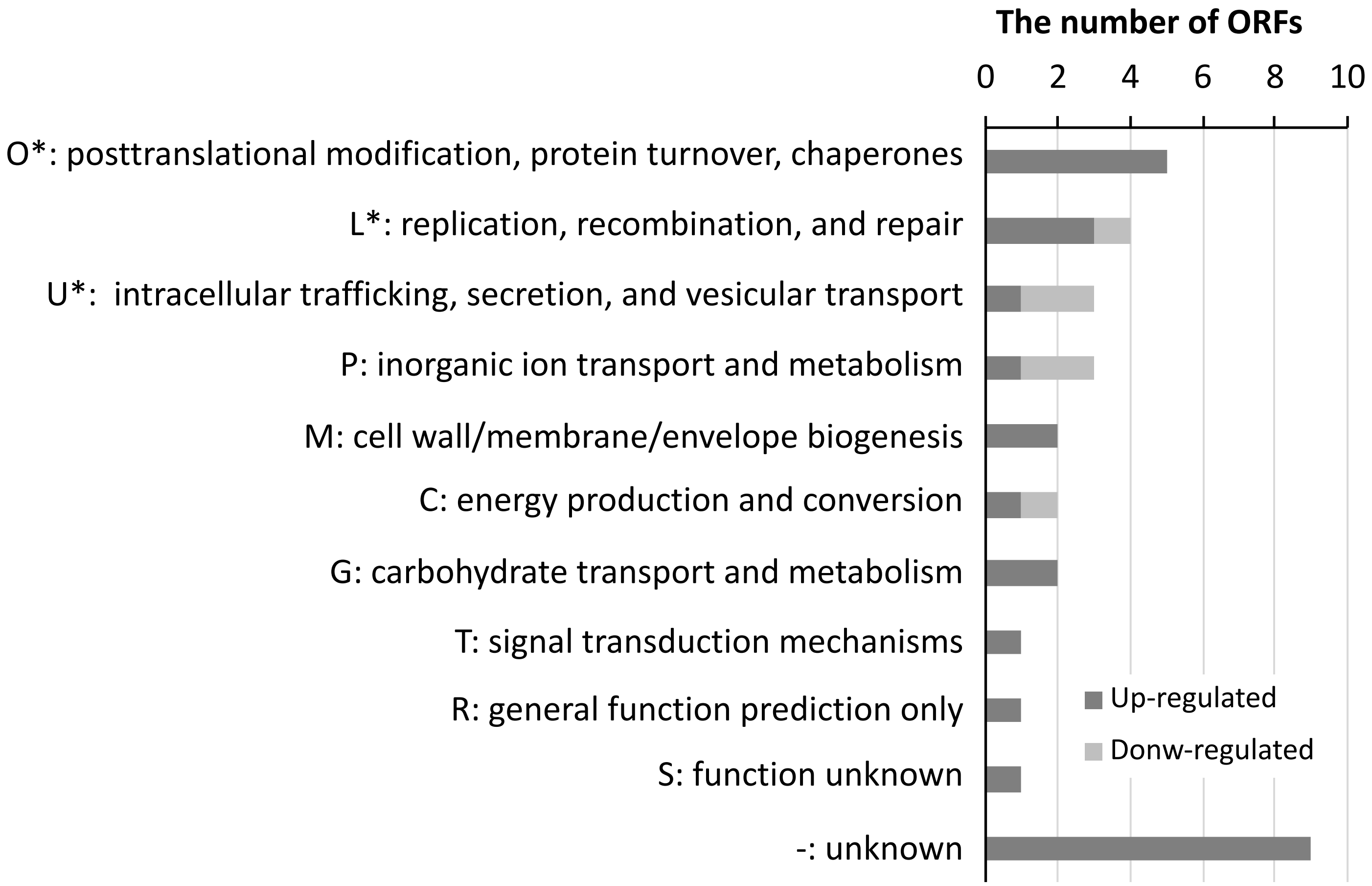

Figure S10. Clusters of Orthologous Groups of proteins (COG) classification of differentially transcribed open reading frames (ORFs) between KT(400) and KT(vc). Asterisks indicate that the relative number of ORFs classified as a given COG code in the differentially transcribed genes was significantly larger than that of ORFs classified as the same COG code among whole-genome ORFs ($P < 0.05$, Fisher's exact test).
